# Evaluation of measles immunity in Turkey: is it still a threat?

**DOI:** 10.3906/sag-1809-54

**Published:** 2019-02-11

**Authors:** Selda KARAAYVAZ, Melahat Melek OĞUZ, Ufuk BEYAZOVA, Gülay KORUKLUOĞLU, Yasemin COŞGUN, Zeliha GÜZELKÜÇÜK, Nur BARAN AKSAKAL, Figen ŞAHİN DAĞLI

**Affiliations:** 1 Division of Social Pediatrics, Department of Pediatrics, Faculty of Medicine, Acıbadem Mehmet Ali Aydınlar University, İstanbul Turkey; 2 Department of Pediatrics, Dr. Sami Ulus Children’s Hospital Research and Education Center, Ministry of Health, Ankara Turkey; 3 Division of Social Pediatrics, Department of Pediatrics, Faculty of Medicine, Gazi University, Ankara Turkey; 4 National Virology Reference Laboratory, Public Health General Directorate Microbiology Reference Laboratory,Ministry of Health, Ankara Turkey; 5 Department of Pediatrics, University of Health Sciences Ankara Children’s Health andDiseases Hematology and Oncology Training and Research Hospital, Ankara Turkey; 6 Department of Public Health, Faculty of Medicine, Gazi University Turkey

**Keywords:** Childhood immunization, measles, measles elimination, measles epidemiology, measles immunization

## Abstract

**Background/aim:**

Measles is one of the important vaccine-preventable diseases with many complications in childhood. This study presents cross-sectional seroepidemiological data, beginning from neonatal cord blood in infants to children under 6 years of age, about waning of measles antibody and tries to suggest the proper time for measles immunization.

**Materials and methods:**

A total of 564 blood samples consisting of neonatal cord blood and samples taken from infants and children at ages of 6, 9, 24–48, and 49–72 months were analyzed for measles seropositivity in a period of 6 months.

**Results:**

Measles seropositivity rate was 72.5% in 109 cord blood samples, 2.6% in 117 infants of 6 months of age, and 3.6% in 111 infants of 9 months of age. Seropositivity was determined in 118 children at 24–48 months and in 109 children at 49–72 months and was 80.5% and 66%, respectively (P = 0.001). These children were vaccinated in the 12th month.

**Conclusion:**

Though measles immunization coverage is 97% in Turkey, population immunity is somewhat lower than expected. Increases of measles cases in Europe and the refugee problem in the country could easily lead to outbreaks. Implementing the first dose of the immunization at 9 months may be an option.

## 1. Introduction

Measles is an important cause of morbidity and mortality in children. Newborns are protected for some time by a specific antibody transferred from their naturally infected or previously vaccinated mothers, but as the antibody starts to decrease after birth these infants become vulnerable to measles [1–3]. However, regional elimination or global eradication of measles is possible through proper vaccination strategies and surveillance of the disease. The World Health Organization has declared measles as the third goal among eradicable infectious diseases [4]. To achieve the highest antibody response in measles immunization in infants, antibody decrease dynamics should be well known. The goal of immunological response and the disease risk should be balanced and measles vaccination strategies should be developed accordingly, as inhibiting the viral circulation becomes impossible when the number of vulnerable individuals increases in a community. Pertaining to our geography, with the ongoing war very close to Turkey’s borders and mass movement of unvaccinated refugees, especially from Syria, there is a much bigger risk of epidemics with vaccine-preventable diseases. Lately increasing measles cases in Europe and vaccine refusals in the country are also adding serious risks. 

Seroepidemiological studies help to determine the amount and duration of natural or vaccine-induced antibodies and their preventive effects on diseases; thus, they support surveillance and vaccine coverage studies for the goals of control and elimination of vaccine-preventable diseases. These studies are indispensable for establishing disease seroprevalences in vulnerable age groups, predicting the need for vaccine campaigns or extra doses beyond national vaccine schedules and the effect of such measures on population immunity, estimating the reasons for epidemics, and setting vaccine coverage goals [5–8].

This study was planned to present cross-sectional seroepidemiological data, beginning from neonatal cord blood in infants to children under 6 years of age, about the waning of measles antibody and to suggest the proper time for measles immunization.

## 2. Materials and methods

This study was conducted in a period of 6 months with 564 healthy children in the Gazi University Medical School Hospital in the capital city of Turkey. The study’s neonatal arm comprised cord blood samples of newborns whose mothers gave consent before delivery. A group of infants and children who had been brought for well-child visits and had no immunological problems or natural measles infection history were recruited after obtaining parental consent. All immunization documents were checked. Recruited infants younger than 12 months of age were not vaccinated for measles, while those older than 12 months were vaccinated one time. Cord blood samples were collected in the delivery room and the rest in the well-child clinic after obtaining a detailed health history and physical examination of the child**.**

After collecting blood samples of 5 mL, centrifugation was completed immediately and serum samples of 3 mL were stored in a freezer at –70 °C in the hospital’s microbiology laboratory. After terminating the study, all samples were transferred to the Ministry of Health’s Public Health General Directorate Microbiology Reference Laboratory and National Virology Laboratory for testing. Before testing, all samples were dissolved and processed at room temperature. The analysis was performed with an Enzygnost Anti-Measles Virus/IgG kit (Siemens, Marburg, Germany) according to the manufacturer’s protocol. A value of ΔA OD of <0.100 was accepted as anti-measles IgG-negative while ΔA OD of >0.200 was anti-measles IgG-positive and values between 0.100 and 0.200 were anti-measles IgG-intermediate.

The study protocol was approved by the local institutional ethics committee. 

### 2.1. Statistical analysis 

IBM SPSS version 21.0 (IBM Co., Armonk, NY, USA) was used. For the categorical data, descriptive analysis was performed and presented as number and percent values. For comparisons chi-square and Fisher’s exact test were used and P < 0.05 was accepted as significant. As the anti-measles intermediate IgG values were very close to the negativity limits, they were consequently accepted as negative. 

## 3. Results 

A total of 564 infants and children were evaluated; age and sex distributions for the entire group are shown in Table 1. There were no statistically significant differences according to the sex or age distributions (P > 0.05). Comparison of measles seropositivity rate among sexes and age groups is shown in Table 2. 

**Table 1:** Age and sex distribution of the study group.

	N	Female (n) (%)	Male (n) (%)
Cord blood	109	61(56)	48 (44)
6 months	117	46 (39.3)	71(60.7)
9 months	111	61 (55)	50 (45)
24–48 months	118	50 (42.4)	68 (57.6)
49–72 months	109	60 (55)	49 (45)
Total	564	278 (49.3)	286 (50.7)

**Table 2:** Comparison of measles antibody seropositivity levels of sexes among different age groups.

Age	Females (n) (%)	Males(n) (%)	P
Cord blood	46 (75.4)	33 (68.8)	0.519
6 months	0 (0)	3 (4.2)	0.278
9 months	2 (3.3)	2 (4)	1.00
24–48 months	39 (78)	56 (82.4)	0.640
49–72 months	39 (65)	33 (67.3)	0.841
Total	146 (52.5)	127 (45.7)	>0.05

P < 0.05 is significant.

Measles seropositivity rate was 72.5% in cord blood samples (n = 109), 2.6% in infants who were 6 months of age (n = 117), and 3.6% in infants who were 9 months of age (n = 111). In 118 children of 24–48 months and in 109 children of 49–72 months of age, who were vaccinated only once at 12 months, the seropositivity rate was 80.5% and 66%, respectively (Figure). 

**Figure 1:**
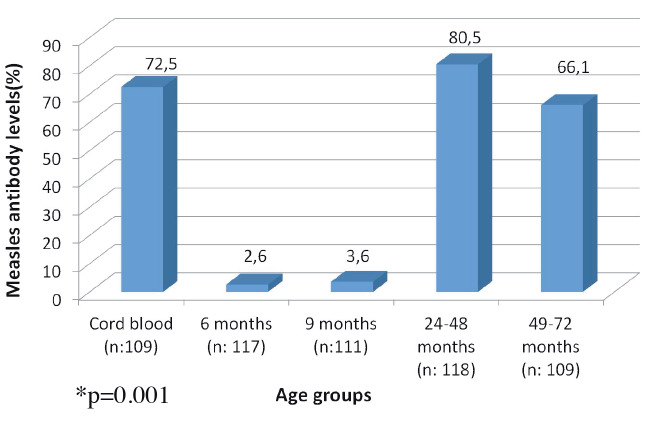
Measles antibody levels of the study group among ages. *P = 0.001.

When all the age groups were compared with each other, statistically different seropositivity levels (P = 0.001) were determined due to high levels of measles antibody in newborns and in the 24–48 months of age category. Similarly, the same significant difference was observed in the comparison of all age groups within the same sexes (P < 0.05). 

## 4. Discussion

This study showed a low measles antibody positivity rate of 72.5% in 109 newborn cord blood samples tested. This finding is very low, particularly for a country with low endemicity. It is well known that newborns already having maternal measles antibody could also experience severe disease when faced with a heavy measles load as much as nonimmune neonates [9]. In a recent study, measles antibody levels of neonates were compared among countries and reported as 90% for Spain, 83% for the Netherlands, 91% for Switzerland, and 80% for England [10,11]. The mothers in the current study were expected to have been vaccinated once during childhood, as a second dose of measles immunization was only implemented after 1998 in Turkey. One dose of measles vaccination is not enough for maintaining the personal or population immunity and sufficient antibody transfer to newborns [12]. 

As Turkey has been fighting measles aggressively since 2000, the incidence of measles dropped from 12/100,000 to 0.7/100,000 in 2014 and so contacting the measles virus and reboosting of the previous immunity decreased in the community. Results of a study indicated that not only newborns but also their mothers were vulnerable to the disease as the initial concentration of maternal antibodies in a newborn is strongly correlated with the mother [1]. 

Measles mortality rate is 1%–5% in infants between 6 and 11 months of age, with a higher mortality risk particularly between 6 and 8 months [5,9]. In theory, measles antibody transferred via the placenta to the newborn has the potential to protect the infant during the first 6–9 months [5,9]. It is postulated that antibody transferred from vaccinated mothers remains for 3.5 months, whereas it is 2 months longer if the mother is naturally immune. At the end of 6 months, all become vulnerable [1,13,14]. 

Measles antibody seropositivity was found 2.6% in the 6-months group. The result was low and compatible with the literature. In a study from Belgium, it was stated that in the infants of mothers who had been naturally infected or vaccinated once during childhood, measles antibody seropositivity was found to be 5% and 1% at 6 months of age, respectively [13]. Lu et al. determined that maternally transferred antibody starts to decrease at the third month and mostly becomes negative at the seventh month with a marked decrease in middle or low socioeconomic levels [15].

In order to maintain measles control during this highly vulnerable period, there is a need to achieve and sustain a high population immunity, as measles vaccination in small infants would not work due to immune immaturity and maternally derived antibody [2].

In this study, measles antibody seropositivity was 3.6% in the 9-months group. However, a regional study conducted in 2003 for the same age group of unvaccinated infants in eastern Turkey showed a 50% measles antibody seropositivity [16]. That remarkable difference probably arose from the regional and temporal differences among studies. 

The measles antibody seropositivity in 118 children of 24–48 months old was 80.5% and it was 66.1% in 109 children of 49–72 months old. The decrease in antibody levels was more than expected. Approximately 5% of measles antibody loss is expected 10–15 years after measles vaccination [17]. 

Similarly, in a regional study conducted in Latin America, after determining very low measles seropositivity in vaccinated infants of 12–23 months old, protection and transfer of vaccines and immunization practices were revised [18]. 

Measles immunization programs aim to reach and maintain ≥95% population immunity so as to protect vulnerable groups and decrease the incidence of disease [5,19]. In every community, for all age groups there can be a sensitive pool even when measles seems to be under control [12]. These vulnerable groups exist due to many factors such as vaccination age, transplacentally transferred antibody, effectiveness of vaccine, antibody losses, and quality and regularity of health services, and all have an effect on population immunity balance [20,21]. 

In Turkey, the first dose of measles immunization is implemented at 12 months and the second dose is in primary school, in the first grade. Actual measles immunization coverage is declared to be 97% for 2 doses [22], but unprotected individuals seem to be much more than expected. Newborns and their mothers, infants under 12 months of age, and preschool children seem to be at risk. In the first half of 2018, measles cases indicated a 10-fold increase in Turkey, rising from 9 cases to 85. The predicted number was 200 cases by the end of the year. 

Mass movement of the 3 million Syrian refugees to the country also poses a risk as 1/3 of this population are children and only 200,000 are harbored in regular camps with access to regular public health services and the rest of the immigrant population resides widely scattered in certain cities (http://multeciler.org.tr/turkiyedeki-suriyeli-sayisi/). Moreover, childhood vaccine refusals are increasing quickly in our community, parallel to the world, and reached 24,000 cases in the last year.

The World Health Organization’s Regional Office for Europe calls on all countries to immediately implement broad, context-appropriate interventions to stop the further spread of measles and outbreaks as over 41,000 children and adults were infected in the region in the first half of 2018 with 37 deaths (http://www.euro.who.int/en/home). 

In conclusion, although measles immunization coverage is 97% in Turkey, population immunity is somewhat lower than expected. Increase of measles cases in Europe and vaccine refusals in the country together with the refugee problem could easily lead to outbreaks. Implementing the first dose of immunization at 9 months can be an option but more data are needed to decide on the most appropriate immunization schedule for measles in Turkey.
